# The efficiency of single-session photobiomodulation on healing of periapical bone lesions using CBCT: a randomized clinical trial

**DOI:** 10.1007/s10103-024-04242-5

**Published:** 2025-01-23

**Authors:** Hend Hamdy Ismail, Maram Farouk Obeid, Ehab ElSaid Hassanein

**Affiliations:** Faculty of Dentistry, Ain Shams University, Organization of African Unity Street, Cairo, Egypt 11766

**Keywords:** Soft tissue laser, Photobiomodulation, PBM, Bone healing, Periapical lesion, CBCT, Randomized clinical trial

## Abstract

As photobiomodulation is growing in the dental field the aim of this prospective, two-arm clinical trial was to assess the radiographic changes for chronic periapical bone lesions related to mandibular molars after primary root canal therapy with or without applying Diode laser on soft tissue. The samples were randomly divided into a Laser group and a mock laser (ML) group. Preoperative CBCT images were compared 12 months later with postoperative CBCT to gauge the changes in the volume of the bony lesion by two observers. The kappa coefficient was calculated to assess the intra-observer agreement. Data were presented as median, mean, and standard deviation (SD) values. For parametric data (age), a Student’s t-test was used. Qualitative data (Gender) was presented as frequencies and percentages and then analyzed by Chi-square test. The Wilcoxon signed-rank test was used to compare CBCT measurements pre- and post-treatment. The significance level was set at *P* ≤ 0.05. The collected data in this study showed that there was no statistically significant difference between median volumes of the periapical lesion in the Laser and ML groups (P-value = 0.564, Effect size = 0.237) and (P-value = 0.452, Effect size = 0.310), respectively while, there was a statistically significant decrease in the volume of periapical lesion post-treatment within each group (P-value < 0.001). The size of the periapical bony lesion decreased significantly after conventional endodontic treatment whether Laser was applied or not. The study protocol was enrolled as NCT04311905 at www.clinicaltrials.gov database on (17/03/2020) after the approval of the Ethics Committee, Faculty of Dentistry, Ain Shams University (FDASURecID091703).

## Introduction

Chronic periapical bony lesions are primarily caused by microorganisms of endodontic origin and their byproducts [1]. This condition is frequently the result of an inflammatory immune reaction, which can be identified by periapical radiography [2]. Effective treatment of root canal infections is essential for repairing periapical lesions and reducing associated inflammation, typically addressed through conservative orthograde endodontic treatment [3]. Success in this treatment relies heavily on proper disinfection of the root canal spaces through mechanical preparation and the use of appropriate irrigating solutions to reduce bacterial presence [4]. This conservative therapy can result in complete to partial apical healing of lesions, with a success rate of up to 86% [5]. Consequently, there is intense debate on how to enhance healing kinetics to improve recovery and prognosis [6].

Since its development by Maiman in 1960, laser technology has been frequently used in dentistry [7]. An area of growing interest is photobiomodulation (PBM) or low-level laser therapy (LLLT), which has gained attention due to its ability to penetrate tissues, promote healing, and reduce pain without significant side effects [8]. PBM uses a red or near-infrared laser with an energy output of up to 500 mW and a wavelength range of 600–1000 nm [9–11]. In endodontics, the key element of PBM is its ability to stimulate tissue repair through cellular biostimulation [12]. This process does not heat soft tissue; instead, the energy from absorbed photons generates photochemical, photophysical, and photobiological effects [13]. When an appropriate dose of laser light interacts with cells, functions such as mitochondrial ATP synthesis can be enhanced, accelerating proliferative processes [14]. Moreover, this coherent light beam can increase local circulation and collagen synthesis [15]. Previous ex-vivo investigations have demonstrated that low-power lasers significantly impact osteoblast differentiation, proliferation, and calcification [16]. Additionally, it has been shown to promote bone formation after tooth removal by creating local conditions that accelerate bone growth and edema resolution [17].

Few in-vivo investigations have highlighted the benefits of PBM in the healing of periapical bone lesions in molars. This study aims to assess the outcomes for a group of patients with chronic periapical bone lesions related to mandibular molars who underwent primary root canal therapy with or without Diode Laser application. This prospective, two-arm, non-significant-risk clinical study evaluated the healing of periapical lesions by comparing the volume of the bony lesion on CBCT images taken before and 12 months after conventional root canal treatment. The null hypothesis posits that there is no discernible difference in the healing process when Laser is applied.

## Methods

### Study aim, design, and setting

The aim was to assess the outcomes for a group of patients with chronic periapical bone lesions; related to mandibular molars; who underwent primary root canal therapy with or without diode Laser by measuring the size of the bone defect before and after interventions on CBCT.

**Fig. 1:**
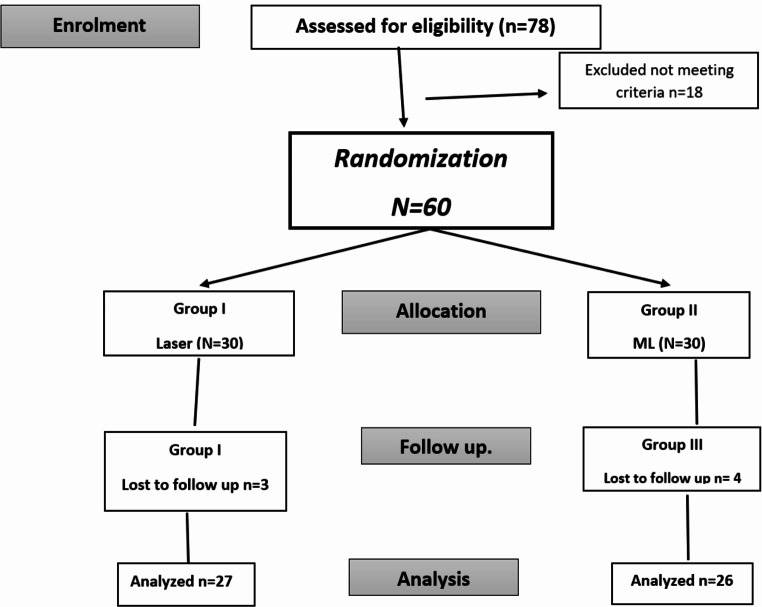
Study CONSORT flow diagram

The design of this study adheres to the Consolidated Standards of Reporting Trials declaration CONSORT [18]. It is a prospective, two-arm, non-significant risk clinical, single-blinded, randomized, placebo-controlled clinical trial. The subjects were classified as presented in Fig. 1.

### Ethics and consent to participate

With the identifier NCT04311879 (17/03/2020), the research protocol was enrolled into the clinical trials database at www.clinicaltrials.gov. The Ethics Committee of the Faculty of Dentistry of Ain Shams University reviewed it and gave its approval (Approval code: FDASURecID091703). All methods were performed per the relevant guidelines and regulations of the Consolidated Standards of Reporting Trials declaration CONSORT [18].

Participants for the study were selected from the outpatient endodontic clinic at the Faculty of Dentistry at Ain Shams University, between July 2017 and January 2020. Each applicant was given a detailed explanation of the study’s objectives, methodology, benefits, and potential risks. All participants who were included in the study signed a consent form.

### Sample size calculation

This was estimated with referral to calculations and guidance of data from an earlier study [19]. It was estimated using Power 3 and it was built on the healing assessment of periapical lesions utilizing CBCT after one year. The alpha error was set at 0.05 and the beta error at 0.1 (power 90%). The sample size was 27 patients per group and shifted to 30 to compensate for the dropouts. The sample size was calculated on IBM SPSS Statistics (Windows, Version 23.0. Armonk, NY: IBM Corp).

### The characteristics of participants (eligibility criteria)

The participants in our study were healthy individuals between the ages of 25 and 40 who had one radiographic apical radiolucency (less than 1500 mm^3^) related to mandibular permanent teeth. Periapical radiographs were done to exclude teeth with immature apex, root fracture, prior root canal, or required considerable prosthetic rehabilitation. Additionally, patients with widespread periodontal disease, pregnant or nursing women, and those with a pre-existing medical condition or dental issue that put them at risk during the experiment or those with any systemic diseases that may affect the wound-healing process, as well as heavy tobacco users, were also banned from participation.

### Diagnosis and patient acceptance

An experienced endodontist (primary researcher) carried out all aspects of dental management. After detailed medical and dental history, clinical and radiographic assessment was done to establish an accurate diagnosis. The presence of periapical lesions was documented by having a preoperative CBCT imaging (90 kVp, 12 mA) applying 0.75 mm^3^ voxel size. The time of exposure was 26 s.

Out of the 78 patients initially considered for the study, only 60 were included. The remaining 18 patients were excluded due to various reasons: 6 patients had underlying systemic health problems, such as uncontrolled diabetes, which could affect the healing process; 3 patients were heavy smokers; 2 patients had previously undergone root canal treatment on the targeted teeth; 2 patients refused to participate; and 1 patient was excluded due to pregnancy.

### Root canal treatment

Under rubber dam isolation, endodontic access was made after the administration of local anesthesia (1:80,000 Arcaine, Aarge Pvt Ltd., India). The working length estimated by an apex locator (Root ZX mini, J. Morita, Japan) was verified by a periapical radiograph. The rotary nickel-titanium Protaper Gold system (Dentsply Maillefer, Ballaigues, Switzerland) was used to mechanically prepare canals by the manufacturer’s instructions. Depending on the canal’s original diameter, finishing files ranging from F1 to F5 mounted on a torque-controlled endodontic motor (X-Smart; Dentsply Sirona) were used. A 27-gauge needle attached to a 3 mL syringe (Kendall, Covidien, Mansfield, MA, USA) was used to irrigate between the files. The irrigation solution used was 3 mL of 2.5% NaOCl. This was followed by a final rinse of 3 mL of 2.5% NaOCl, 3 mL of EDTA 17% (Denteck, Zoetermeer, Netherlands), and 3 mL of ordinary saline in between and at the end before filling the canal.

All canals were dried out with paper points and packed with gutta-percha and AH Plus sealer (Dentsply Maillefer, Ballaigues, Switzerland) applying the warm vertical condensation technique. All teeth were restored with composite resin (Dentsply Sirona, Charlotte, NC, USA) at the same visit. A dental operating microscope (Carl Zeiss, Jena, Germany) X8 magnification was used throughout the treatment.

### Randomization

Following that, patients were divided into two groups (*n* = 30) based on how the diode laser was used. The researcher made envelopes with secret group codes progressively distributed to the qualified patients. To have sufficient randomization utilizing an equal proportion randomization allocation ratio, the operator opened the envelopes and logged the codes with the appropriate patient characteristics on a computer. The patient was blinded to the group he or she enrolled in.

### Intervention

- *In the Laser group*, the primary researcher positioned the laser applicator over the oral mucosa with the beam concentrated apically. The PBM Parameter is presented in Table 1 [20–22].
- *In the ML group*, the diode laser tip was positioned similarly as in the Laser group but without laser activation by the same researcher.

**Table 1:** PBM parameters

Parameter	Laser Group	ML Group
Manufacturer	LITEMEDICS	
Model identifier	Serial Number: 148, SwvM. 150VS108VT.100	
wavelengths	970 nm	
Pulse mode	Continuous, 10 Hz	
Beam Spot size at target	Around 0.3 mm	
Exposure Duration	60 s per point	
Number of points irradiated	Two points at the level of the targeted root apex buccally and lingually	
Area irradiated	Around 0.7 mm2 (for both points)	0 (device turned off)
Application technique	Without movement in a contact mode	
Power:	0.5 W	0 W (device turned off)
Energy per Point:	30 J	0 J (since the laser is off)
Total irradiation Energy:	60 J	0 J (since the laser is off)
Power Density:	2.86 W/cm²	0 W/cm²
Total energy	60 J	0 J
Number & frequency of treatment sessions	Single session immediately after RCT	

### Recall rate of patients

Each participant was recalled after one year, the recall rate for this study was 88.3%. Out of 60 patients who got treatment, 4 patients in the ML group and 3 patients in the Laser group with a total of 7 patients did not return owing to personal reasons, thus, 53 patients returned for follow-up care and undertook post-operative CBCT using the same machine and the same parameters used in the preoperative scan.

### Assessment of periapical Healing

Two different, skilled examiners, who were concealed from the groups, performed the measures to guarantee their reliability using Planmeca software (Planmeca Romexis^®^; Planmeca). All CBCT pictures were analyzed on a 27-inch flat-panel computer display with a pixel resolution of 2.560 × 1.440. Brightness and contrast were the only adjustments made after the filters were set to normal.

For both the preoperative and postoperative CBCT scans, the volume of the periapical lesion was measured in mm^3^. The investigator manually traced the shape of the lesion in the axial cut using the Romexis software’s manual segmentation application, repeating the process every 2 mm, until the lesion vanished from the bone and healthy bone was visible. The lesion volumetric region was then determined by selecting “create region” in the application. The software calculated all the data.

### Statistical analysis

The kappa coefficient was calculated to assess the agreements between the observers. The numerical data was explored for normality by checking the distribution of data and using tests of normality (Kolmogorov-Smirnov and Shapiro-Wilk tests). Age data showed a normal (parametric) distribution while CBCT data showed non-normal (non-parametric) distribution. Data were presented as median, mean, and standard deviation (SD) values. For parametric data, Student’s t-test was used for comparisons between age values in the two groups. For non-parametric data, the Mann-Whitney U test was used to compare between the two groups. The Wilcoxon signed-rank test was used to compare CBCT measurements pre- and post-treatment. Qualitative data (Gender) was presented as frequencies and percentages. The chi-square test was used for comparisons between the groups. The significance level was set at *P* ≤ 0.05. Statistical analysis was performed with IBM SPSS Statistics for Windows, Version 23.0. Armonk, NY: IBM Corp.

## Results

Out of 78 patients, 60 were deemed qualified after being evaluated according to the predefined inclusion criteria. All participants were recruited after one year, 3 patients from the Laser group and 4 in the ML group failed to follow up, resulting in a final radiographic assessment of 53 patients (27 in the Laser group and 26 in the ML group). None of the participants showed any signs/symptoms of failure clinically.

The agreement for volumetric measurements between observers was found to be excellent (kappa > 90).

Regarding the demographic data (summarized in Table 2), there was no statistically significant difference between mean age values in the two groups. As regards gender; the Laser group showed a statistically significantly higher prevalence of males and a lower prevalence of females than the ML group.

**Fig. 2:**
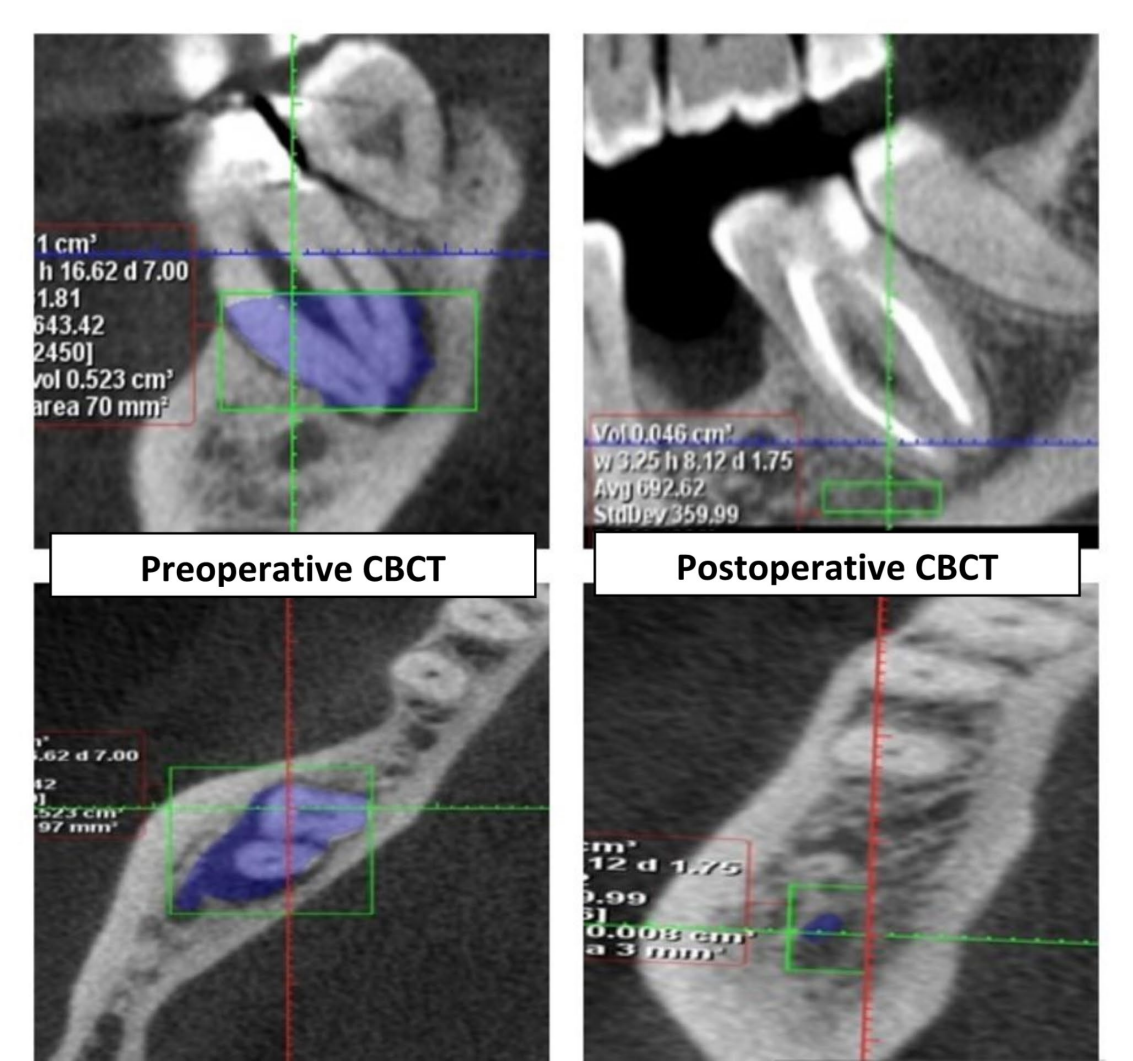
CBCT images showing the difference in the volume of the periapical lesion in the Laser group

**Fig. 3:**
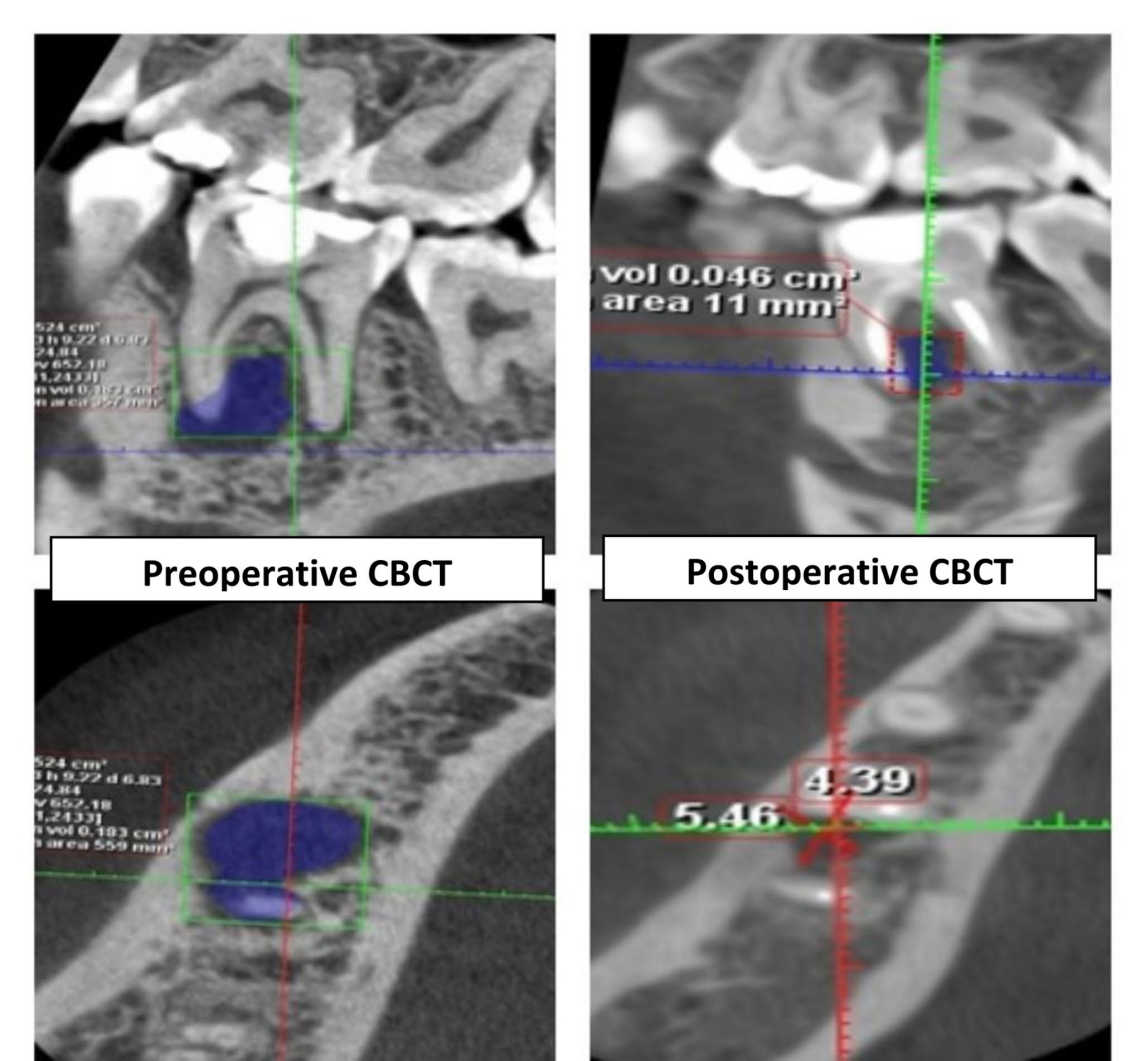
CBCT images showing the difference in the volume of the periapical lesion in the ML group

Regarding the comparison between the two groups (presented in Table 3; Figs. 2 and 3); there was no statistically significant difference between median volumes of the periapical lesion in the two groups before treatment (P-value = 0.564, Effect size = 0.237). Also, after the intervention, there was no statistically significant difference in the groups (P-value = 0.452, Effect size = 0.310).

Regarding the changes within each group (presented in Table 3); there was a statistically significant decrease in the volume of periapical lesions post-treatment in each group (P-value = 0.002, Effect size = 0.883) and (P-value = 0.002, Effect size = 0.884), respectively.

**Table 2:** Mean, standard deviation (SD), frequencies (n), and percentages (%) of demographic data in the two groups

Demographic data	Laser Group (*n* = 27)	ML Group (*n* = 26)	*P*-value
Gender [n (%)]			0.001*
Male	**21 (77.8%)**	**14 (53.9%)**	
Female	**6 (22.2%)**	**12 (46.1%)**	
**Age (Years)**			
**Mean (SD)**	**38 (9.6)**	**37.6 (9.5)**	**0.826**

*: Significant at *P*≤0.05

**Table 3:** Comparison between the volume of the periapical lesion (mm^3^) in the two groups

Time	Laser Group	ML Group	*P*-value	Effect size (d)
Pre-treatment				
Median	**50.5**	**77**	**0.564**	**0.237**
Mean (SD)	**160.1 (311.8)**	**91.4 (72.8)**		
Post-treatment				
Median	**19**	**10.5 (2–83)**	**0.452**	**0.310**
Mean (SD)	**78.4 (155)**	**20 (23.3)**		
*P*-value	0.002*	0.002*		
*Effect size (r)*	**0.883**	**0.884**		

*: Significant at *P*≤0.05

## Discussion

PBM is a type of phototherapy in which a monochromatic, coherent light of low intensity is used. In several fields of medicine and dentistry, it has emerged as a viable therapy option. Even though there are numerous studies demonstrating the benefits of PBM [7–9], its impact on periapical lesion healing is not clear. Thus, we aimed in our clinical study to test the effects of using Laser following conventional endodontic therapy on bone healing in terms of volume measured by CBCT in mandibular molars with periapical lesions.

This study is a randomized clinical trial (RCT); the most effective resource in evidence-based medicine. It was chosen as it accurately reflects the clinical aspect with the highest dependability [23].

It is known that the primary goal of photobiomodulation (PBM) is to achieve therapeutic effects without inducing thermal damage to tissues (non-thermal biostimulation) [10]. In our investigation, we chose a Diode laser with 970 nm wavelength. This wavelength is efficiently absorbed by water and hemoglobin, which facilitates increased local circulation, enhanced oxygen delivery, and improved cellular metabolism [24]. While other wavelengths (e.g., 660 nm, 810 nm, 1064 nm) have also been shown to produce biostimulatory effects, the choice of wavelength often depends on the desired depth of tissue penetration. For instance, the 660 nm wavelength is effective for superficial tissue applications, whereas wavelengths in the near-infrared range, such as 810 nm and 980 nm, are better suited for deeper tissue penetration, which is necessary for the studied effect, as the laser aims to impact bone repair in the periapical area [25]. The laser beam was targeted over the alveolar mucosa at a location corresponding to the mesial and distal apices of each tooth both buccally and lingually to be aligned with previous studies [20–22, 26]. Given that it enables blinding in clinical research, a mock laser was used to serve as a placebo in this case [27]. The healing of periapical bone lesions was measured in terms of mm^3^ on CBCT as it is proved to be more accurate at detecting this than digital periapical radiographs in the long-term assessment of root canal treatment success [28]. Furthermore, CBCT was quite accurate in determining the volume of periapical pathosis [29].

Our null hypothesis was accepted, and the results showed that there is no discernible difference in the healing process when Laser is applied. The size of the bony lesions decreased after conventional root canal treatment, and this was in line with a previous study [30]. Our demographic data revealed the significant presence of males in the Laser group. However, it was previously concluded that gender had no significant effect on bone healing [31–33].

While we recognize the importance of comparing our findings with previous studies, it is important to note that there is a lack of articles specifically investigating bone healing after endodontic treatment with photobiomodulation (PBM). As a result, direct comparisons with other studies are not feasible. However, formerly, it was demonstrated that PBM is effective in wound healing [34] as well as bone healing [35]. It could improve callus formation in the early phase of the bone healing procedure and may speed up bone growth and edema resolution [17, 36]. This positive effect of PBM marked by decreasing the volume of the bony lesion can be credited to the impact of the laser beam [8, 37, 38]. Usually, this leads to non-thermal effects with an increase in the generation of ATP, the “energetic currency” for cells, and accelerates cells’ different functions [14]. Additionally, PBM is recognized as a non-invasive kind of light therapy (phototherapy) [16]. It affects cellular metabolism as well as local microcirculation [15]. It increases prostaglandin synthesis providing potent anti-inflammatory effects [39]. Likewise, PBM promotes oxidation/reduction in cells by activating the mitochondria, leading to enhanced ATP generation and protein synthesis, which can hasten periradicular healing after root canal treatment [40]. All these biological processes may account for the promising effect on healing as reported earlier [34–36, 41].

The statistical lack of significance between the Laser and ML groups in our outcomes might be attributed to several factors. Firstly, the timing of the post-treatment evaluation may have impacted the results; our 12-month follow-up period may not have been sufficient to capture the full extent of the healing process facilitated by PBM. Longer follow-up periods are often necessary to observe significant differences in tissue healing and regeneration​ [25]. Moreover, the mode of laser application in this trial could also play a role. The laser was used only once immediately following endodontic therapy. In the literature, there is a wide range of PBM protocols for bone healing; from daily to alternate-day administration for 15–21 days [42]. This study used a single session as per the protocols followed in previous investigations [21, 43, 44]. However, this can be considered a limitation of this study. As a previously published systematic review [9] suggests, more research needs to be conducted with varied application protocols to validate the effectiveness and potential risks associated with PBM in endodontics. Nonetheless, it can be an effective complementary tool to traditional treatment.

After all, there is a terrific demand to establish an efficient protocol of laser use concerning wavelength, power output, and application approaches to identify the best possible protocol to use diode laser or PBM as an aid in healing. Our study contributes to the limited body of research exploring the impact of PBM on periapical bone healing in the context of endodontic treatment. Future research should aim to fill this gap by conducting more targeted studies on the specific effects of PBM on periapical bone healing following endodontic therapy. To enhance the understanding of PBM’s impact on periapical healing, we recommend implementing shorter review intervals in study designs. Furthermore, utilizing more sophisticated methods, such as volumetric measurements or established CBCT periapical indices, will yield a more comprehensive and precise evaluation of healing outcomes. Lastly, we advocate for the exploration of AI-based software in conjunction with these traditional methods to improve lesion measurement and assessment.

## Conclusion

The size of the periapical bony lesion decreased significantly after conventional endodontic treatment whether Diode Laser is applied or not.

## Data Availability

The data presented in this study are available upon request from the corresponding author.

## Abbreviations

PBM: Photobiomodulation
CBCT: Cone beam Computed tomography
ML: Mock Laser
ATP: Adenosine Triphosphate
